# Self-reported data in environmental health studies: mail vs. web-based surveys

**DOI:** 10.1186/s12874-019-0882-x

**Published:** 2019-12-12

**Authors:** Manuella Lech Cantuaria, Victoria Blanes-Vidal

**Affiliations:** The Maersk Mc-Kinney Moller Institute, University of Southern Denmark, Campusvej 55, 5230 Odense M, Denmark

**Keywords:** Survey mode, Data collection, Survey design, Questionnaire, Mixed-mode surveys, Rural residents, Mail survey, Web survey

## Abstract

**Background:**

Internet has been broadly employed as a facilitator for epidemiological surveys, as a way to provide a more economical and practical alternative to traditional survey modes. A current trend in survey research is to combine Web-based surveys with other survey modes by offering the participant the possibility of choosing his/her preferred response method (i.e. mixed-mode approach). However, studies have also demonstrated that the use of different survey modes may produce different responses to the same questions, posing potential challenges on the use of mixed-mode approaches.

**Methods:**

In this paper, we have implemented a statistical comparison between mixed-mode survey responses collected via mail (i.e. paper) and Web methods obtained from a cross-sectional study in non-urban areas of Denmark. Responses provided by mail and Web participants were compared in terms of: 1) the impact of reminder letters in increasing response rates; 2) differences in socio-demographic characteristics between response groups; 3) changes on the likelihood of reporting health symptoms and negative attitudes towards environmental stressors. Comparisons were mainly performed by two sample t-test, Pearson’s Chi-squared test and multinomial logistic regression models.

**Results:**

Among 3104 contacted households, 1066 residents decided to participate on the study. Out of those, 971 selected to respond via mail, whereas 275 preferred the Web method. The majority of socio-demographic characteristics between these two groups of respondents were shown to be statistically different. The use of mailed surveys increased the likelihood of reporting health symptoms and negative attitudes towards environmental stressors, even after controlling for demographic characteristics. Furthermore, the use of reminder letters had a higher positive impact in increasing responses of Web surveys when compared to mail surveys.

**Conclusions:**

Our main findings suggest that the use of mail and Web surveys may produce different responses to the same questions posed to participants, but, at the same time, may reach different groups of respondents, given that the overall characteristics of both groups considerably differ. Therefore, the tradeoff between using mixed-mode survey as a way to increase response rate and obtaining undesirable measurement changes may be attentively considered in future survey studies.

## Background

Surveys are widely used as a data collection method in social sciences and medical research. The most traditional survey administration approaches are paper-based, telephone or face-to-face surveys. However, the application of these survey modes usually involves a high amount of resources and time, resulting in elevated costs and work demand. The use of Web-based surveys brings a number of advantages in comparison to the most traditional approaches, such as cost-effectiveness, easiness of implementation, coding and data storing, and possibility of data encryption as a protection measure [1]. Moreover, internet access has rapidly increased during the last decades and, therefore, Web questionnaires are nowadays the most employed survey mode in quantitative research worldwide [2].

A growing body of literature has investigated the use of Web-based surveys in population studies in comparison to more traditional methods. A large part of these studies have focused on the comparison of Web surveys with telephone and/or face-to-face approaches [3–6]. These data collection methods, however, present substantial differences in many aspects (e.g. personal contact between the study participant and interviewer, flexibility of the participant when answering the questionnaire and participant’s perception of his/her level of anonymity) which makes their comparison even more challenging [7]. Comparing conventional mail surveys and Web-based surveys, on the other hand, is easier, due to a number of similarities between these modes.

There are several studies focused on the comparison between mail and Web methodologies for collecting responses in different study setups. Smyth et al. (2010) [8], for instance, investigated the challenges involved when internet is used to survey small towns and rural communities in the United States, and assessed its effectiveness in comparison to mail surveys. Carini et al. (2003) [9] and Kim et al. (2019) [10] have compared the use of paper and Web-surveys to collect education-related data on 1) college experience and 2) public university and its affiliated health organizations, respectively. Mail and Web surveys have also been explored in different other survey topics, such as household practices [11], consumption habits [10, 12], tourism [7] and medical care experiences [13].

Given the drastic increase in the use of Web surveys, and its potential problems with coverage and nonresponse error, the implementation of different data collection strategies have gained growing attention over the last years. One of these strategies involves the design and implementation of mixed-mode surveys [14]. A mixed-mode design is mainly applied in any of the two data collection phases [15]: 1) in the contact phase, when more than one communication method is used when participant is contacted [13, 16]; 2) in the response phase, when more than one mode is used for the actual collection of data [11, 13].

For the response phase mode change, it is believed that mixing two or more response methods may result in higher response rates since respondents’ preference in relation to survey mode may considerably differ [17]. However, studies have also demonstrated that the use of different survey modes may produce different responses to the same questions, posing potential challenges on the use of mixed-mode approaches [18, 19]. The process of responding a questionnaire comprises numerous steps, which can be summarized as: 1) comprehending the question; 2) retrieving information from memory; 3) analyzing the captured information in relation to the question; 4) providing a response. All these stages involve a number of cognitive reactions from the respondent, which may be influenced by the means in which the survey was conducted, even among respondents with similar characteristics [17, 20].

Differences in responses related to the mode in which the survey was conducted are strongly dependent on the way information was transmitted to participants (i.e. visually, aurally or both). The implementation of modes that involve different transmission mechanisms (e.g. telephone interviews and mail survey, which are exclusively aural and visual, respectively) results in higher measurement errors in comparison to unified mode questionnaires (e.g. mail and Web surveys, which are both visual) [15]. However, mode-related disparity between responses may still exist even when a unified mode design is used. For the case of mail and Web surveys, differences may arise due to variations in e.g. visual/graphical presentation and questions’ structure. Furthermore, responses obtained by these modes may also differ according to the participant’s familiarity with the medium used for surveying [15].

Other strategies to improve participation rates in survey research have been evaluated by numerous studies [21–25], such as reminder letters, pre-notification and monetary incentive. In a systematic review study done by Nakash et al. (2006) [26], the implementation of reminder letters showed the most significant effect on response rates in comparison to questionnaire length, re-ordering of questions and incentives. However, the size of the effect of reminders demonstrated in these studies drastically varies [21], raising the question whether this effect may be related to the mode in which the survey is conducted.

Between October 2015 and March 2016, our research group conducted a cross-sectional survey study focused on the assessment of health and quality of life of residents living in non-urban areas of Denmark. In cross-sectional studies, two mixed-mode designs are most commonly employed. The first one is a simultaneous design, where respondents are offered two or more survey options at the same time, so that the individuals can participate on the study in different ways in accordance to their personal preferences. The second one is a sequential mixed-mode design, where one response mode (usually the least expensive) is offered before the other mode [15]. In our study, we have implemented a simultaneous mixed-mode approach, by offering residents the possibility of answering either a printed or a Web version of the questionnaire. Even though the mail and Web questionnaires follow a unified-mode design and were developed in order to minimize differences between the two modes, it is still not clear whether the responses collected via the two approaches statistically differ.

The purpose of this paper is to carry out a statistical comparison between mixed-mode survey responses collected via mail (i.e. printed) and Web methods. The survey used in this study was conducted in non-urban areas of Denmark as part of a cross-sectional epidemiological study. Most specifically, we aimed to find an answer for the following research questions:

1. Does the distribution of mail and Web responses before and after residents received a reminder letter significantly differ?
2. Are the socio-demographic characteristics of the participants choosing to respond via mail different from those selecting the Web method?
3. Are there any differences on the likelihood of reporting health symptoms and negative attitudes towards environmental stressors (i.e. noise, odor, smoke, dust and vibration) between mail and Web survey respondents?

## Methods

### Study design

**Fig. 1:**
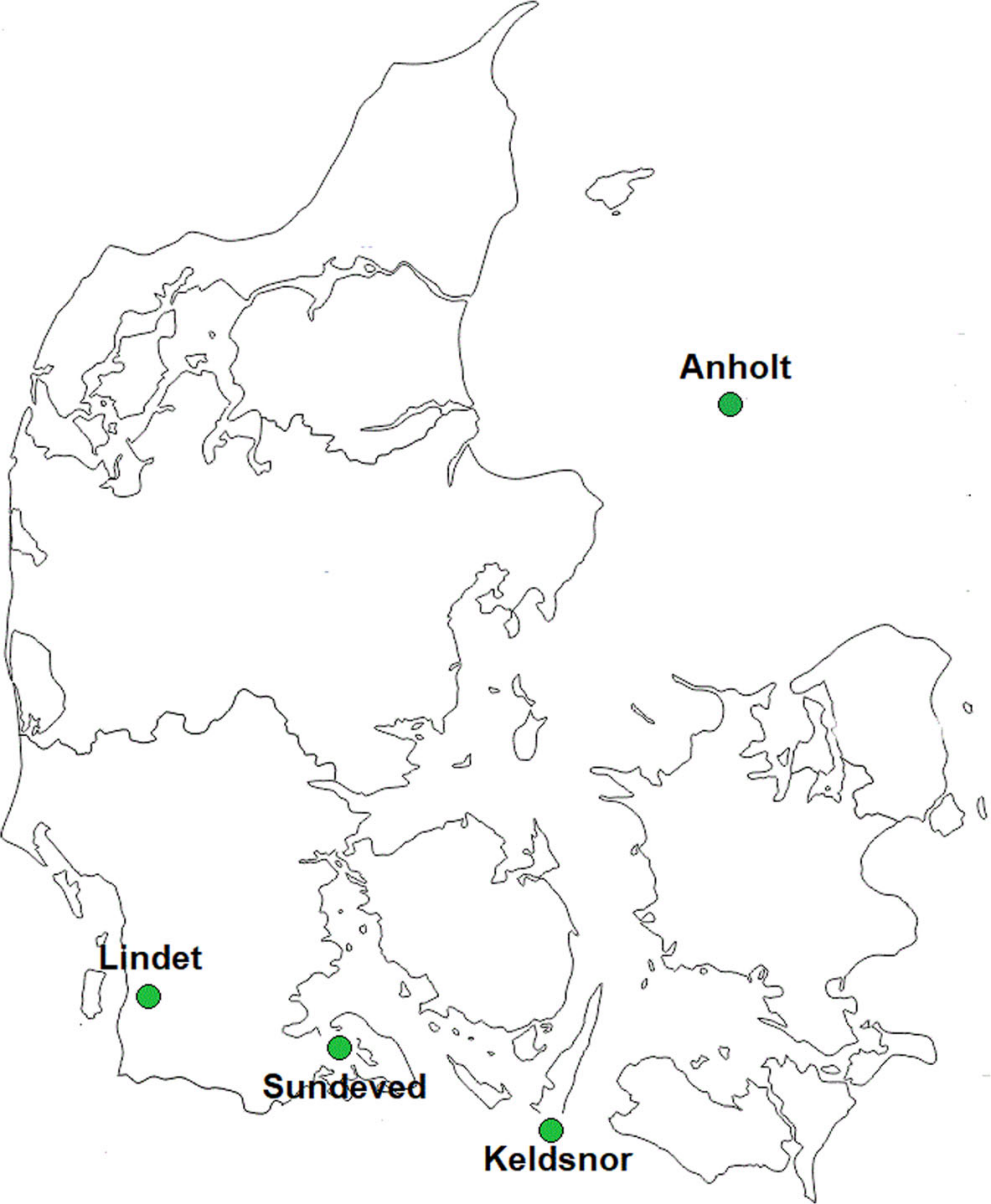
Non-urban areas of Denmark analyzed in this study (Anholt, Lindet, Sundeved and Keldsnor)

A cross-sectional survey was administered to a random sample of non-urban residents of Denmark as a way to assess health and quality of life conditions of residents living close to agricultural and animal production facilities. This study covered four non-urban areas of Denmark: Anholt, Keldsnor, Lindet and Sundeved (Fig. 1), each of them representing different levels of exposure to environmental factors. Addresses within these four regions were provided by the corresponding municipality and, out of those, 3104 randomly selected households (adults > 18 years old) were invited to participate in the study. All households were contacted during the period of October 2015 to beginning of March 2016, when field application of animal slurry is restricted by law in Denmark, so that responses were not influenced by an excessive annoyance to odorous compounds and to other related aspects. Our research was carried out in accordance with principles of the Declaration of Helsinki and registered by the Danish Data Protection Agency (Datatilsynet).

All selected households received an informative letter addressed generically “to the householder”, which contained a brief description of the study and simple instructions specifying that the answers should be given by a single participant and that she/he should be older than 18 years. The letter offered, for those who agreed in being part of the study, two options to answer the survey. The first option was to fill in a printed version of the survey and return it by post using a pre-stamped envelope (i.e. the participants did not have to incur any shipping expenses). Both the printed survey and the envelope were sent together with the informative letter. The second option was to complete an online version of the questionnaire (i.e. Web-based survey). All participants were identified through an anonymous code provided together with the informative letter. The web address (URL) was also included in the informative letter. For those who opted to use the Web survey, the questions could only be accessed after providing a valid identification code.

Reminder letters were sent to those residents who had not responded to the survey after 3 weeks from the moment they were first contacted. The reminder letter offered the two options for the participant to respond the survey. For those who preferred to use the online version, both the URL and identification code were again provided. For those who preferred the mail version, we offered them the option to contact us (via SMS or e-mail) requesting a new copy of the questionnaire and a pre-stamped envelope, in case they did not have them any longer. In total, a maximum of two reminder letters were sent to each resident.

As previously mentioned, the households were randomly approached, which means that the probability of being selected did not depend on whether the household was placed in close proximity to, or far away from, potential sources of environmental stressors. However, this study is still at risk of self-selection bias, since the participants have chosen themselves whether they preferred to respond the Web or the mail survey (i.e. they were not randomly assigned to a group). This may distort the representation of a true population and potentially bias the results from statistical analyses [27].

Smyth et al. (2010) [8] have pointed out that the preference for mail surveys is likely to be more pronounced in rural communities, given characteristics of the region such as lack of good quality internet. On the other hand, it is expected that more isolated regions have also a longer distance to a post office, which would motivate the respondent to use the Web version. We thereby carried out a self-selection bias analysis, in order to evaluate whether individuals living in areas with different levels of urbanization and higher exposures to environmental stressors may have been more likely to select a specific survey mode, by using a Pearson’s Chi-squared test.

Moreover, as the environmental stressors hereby analyzed are strongly related to air pollution sources (e.g. animal/agricultural production facilities and road traffic), we further performed a two-sample t-test to compare the levels of air pollution that the two survey mode samples (i.e. Web survey and mail survey respondents) were exposed to. For this analysis, we used data on three very relevant air pollutants averaged for the 5 years prior to the period when questionnaires were sent: NO_2_ (nitrogen dioxide), PM_2.5_ (fine particulate matter) and NH_3_ (ammonia).

### Survey

Survey items were developed with basis on previously validated questionnaires on indoor climate [28] and health [29]. The questionnaire was divided in three main sections:

- *Background Information*: the first part consisted of socio-demographic questions, such as sex, age, smoking and behavioral habits, education and job situation;
- *Environment*: the second section consisted of questions on self-perception of the environment, including questions on annoyance, health concern and behavioral changes due to environmental stressors (i.e. noise, odor, smoke, dust and vibration);
- *Health and well-being*: the last section was the most extensive one and included questions on the frequency of different health symptoms (e.g. nose, eyes and throat irritation, bodily pain, cough, breathing problems and others), diagnosis of acute and chronic diseases and occurrence of allergies and children’s’ health conditions (in case of children living in the household). Moreover, respondents were thereby asked on their mental health conditions such as depression, stress, anxiety, sleep disturbance, etc.

The estimated time to complete the survey was from 10 to 12 min. Both Web and printed surveys consisted of the same questions and were similarly structured. A non-original version of the questionnaire, being this one translated from Danish to English, was included as a supplementary material (Additional file 1).

### Statistical comparison between mail and web surveys

In this study, we aimed to compare four different aspects of responses obtained by mail and Web surveys:

1. Response rate before and after reminder letters: The analysis of response rate has included the calculation of response rates based on the rules from the American Association for Public Opinion Research [30] for RR2 (Response Rate 2). It means that the number of complete and partial responses were divided by the number of responses plus the number of non-responses. Known ineligible cases (i.e. returned letters due to invalid address and refusals due to e.g. the fact that the house is not used on a daily basis (i.e. vacation house) and rare cases of mental sickness informed by a health care assistant) were not considered in the denominator. We analyzed the number of respondents after they were contacted for the first time and after receiving a reminder letter (either the first or the second reminder) and disaggregated the number of responses by mode of completion (i.e. mail and Web surveys).
2. Socio-demographic characteristics of Web and mail survey respondents: Those were compared using two sample t-test and Pearson’s Chi-squared test.
3. Self-reported health symptoms: Participants were asked to estimate the frequency they experienced various health symptoms within the past 2 years, using a 5-point frequency scale (i.e. 0 = “Never/very rarely”, 1 = “Several times per year”, 2 = “Several times per month”, 3 = “Several times per week” and 4 = “Daily”). The occurrence of health symptom was dichotomized into “low frequency” (5-point frequency scale score = 0) and “increased frequency” (5-point frequency scale score ≥ 1). We further compared the prevalence of “increased frequency” responses obtained for each survey method and used logistic regression models to analyze the odds of mail respondents to report increased frequency of health symptoms in comparison to Web survey respondents.
4. Self-reported attitude towards environmental stressors: The survey contained questions about frequency of perception and annoyance level of participants due to environmental stressors (i.e. noise, odor, dust, smoke and vibration). The frequency of perception of each stressor was dichotomized into “low frequency” (score = 0) and “increased frequency” (score ≥ 1) whereas the annoyance due to each stressor was categorized into “not annoyed” (score = 0) and “annoyed” (score ≥ 1). The overall perception and annoyance variables were created and studied as “perception of/annoyed by two or more stressors” vs. “perception of/annoyed by less than two stressors”. Residents were also asked whether: 1) they were concerned about the adverse health impacts of any of the environmental stressors (i.e. health concern variable, dichotomized into “no” and “yes”); and 2) any of the stressors was interfering in their behavioral habits, by e.g. reducing the frequency of outdoor activities or preventing them to ventilating their houses (i.e. behavioral interference variable, dichotomized into “no” and “yes”). The overall health concern and behavioral interference variables were also created and analyzed as “concerned by one or more stressors” vs. “not concerned by any stressor” and “behaviorally affected by one or more stressors” vs. “not behaviorally affected by any stressor”, respectively.

Crude and adjusted logistic regression models were used to compare the attitude towards environmental stressors reported by mail and Web survey respondents. The latter models were adjusted for the following potential confounders: age, sex, education, region where the individual lives (i.e. Anholt, Keldsnor, Lindet and Sundeved), smoking status, childhood living environment, presence of children at home, period spent outside home and job situation. Smoking status was measured with the question “Do you smoke cigarettes?”, with the following answers: 1) “Yes, every day”; 2) “Yes, but not every day”; 3) “No, but I have smoked before”; 4) “No, I have never smoked”. Childhood living environment was assessed with the question “Where did you grow up?” and the options: 1) “Large town”; 2) “Village”; 3) “Countryside”. Residents were also asked about the number of people that live in their houses besides themselves and what is their age. The answer to that question was used to create the variable “presence of children at home”. To measure the period spent outside home, residents were asked “For the last 12 months, how many days have you stayed away from your house (have not slept at your house)?” with the potential replies: 1) “Less than 14 days”; 2) “14–27 days”; 3) “28–55 days”; 4) “56–111 days”; 5) “112 days or more”. Finally, the following alternatives were given to the participants to determine their job situations: 1) “Self-employed”; 2) “Employee”; 3) “Unemployed”; 4) “Under education”; 5) “Outside the job market”; 6) “Others (e.g. housewives, househusband)”.

All statistical analyses were performed in R 3.1.2 (R Core Team, 2014).

## Results

### Response rate before and after reminder letters

A total of 1066 valid responses were received comprising 971 mail surveys and 275 Web surveys. Out of those, 61% were received just after the first contact (i.e. no reminder letter was sent), whereas 39% decided to participate on the study only after they have received at least one reminder letter. The overall response rate of this study was 34.3% (Table 1). Among the study regions, Keldsnor was the one that presented the highest response rate (37.0%), followed by Sundeved (35.3%), Lindet (31.2%) and Anholt (27.8%).

**Table 1:** Number of responses and its corresponding response rate obtained at each data collection phase (i.e. after the first time participants were contacted, after participants received at least one reminder letter and for the overall study)

Region	Number of mailed letters ^a^	Number of mailed reminders ^a^	Overall responses		Responses after first contact		Responses after reminder		p-value ^e^
			Number of respondents	Response rate (%) ^b^	Number of respondents	Response rate (%) ^c^	Number of respondents	Response rate (%) ^d^	
Anholt	79	59	22	27.8	20	25.3	2	3.4	< 0.001
Keldsnor	165	134	61	37.0	31	18.8	30	22.4	
Lindet	645	526	201	31.2	119	18.4	82	15.6	
Sundeved	2215	1736	782	35.3	479	21.6	303	17.5	
Total	3104	2455	1066 ^f^	34.3	649	20.9	417	17.0	

^a^ Respondents estimated to be ineligible (i.e. returned letters due to invalid address and refusals due to e.g. the fact that the house is not used on a daily basis (i.e. vacation house) and rare cases of mental sickness informed by a health care assistant) are excluded from this number. In total, 75 households were considered ineligible

^b^ Response rate was calculated by dividing the total number of respondents by the number of mailed letters

^c^ Response rate was calculated by dividing the number of respondents after the first contact by the number of mailed letters

^d^ Response rate was calculated by dividing the number of respondents after a reminder letter by the number of mailed reminders

^e^ Pearson’s Chi-squared test of proportions was used to calculate the difference between the response rates for the two contact points

^f^ Besides these surveys, 13 other responses were received without the identification code and two individuals have answered both the mail and the Web version of the questionnaire. These were not considered in this study and therefore, are not considered in this analysis

Besides the overall response rate, Table 1 also shows the number of responses and its corresponding response rate at each contact point (i.e. first contact: when the first letter was sent; and second contact: after a reminder letter was sent). For all regions, except for Keldsnor, the response rate at the first contact was higher than at the second contact (i.e. after the use of reminder letters). The difference between the response rates for the two contact points was statistically significant (*p* <  0.001). Besides, for all of the study regions, the overall response rate has considerably increased after the use of reminder letters. However, this increment was less substantial for Anholt in comparison to the other regions.

The number of overall responses obtained at each contact point (i.e. after the first point in time the individuals were contacted and after they received a reminder letter) was disaggregated by mode of survey completion (Table 2). Most of participants preferred to respond the mail version of the survey (74%). However, the percentage of mail survey participants was higher when residents received the first letter (77%) in comparison to when they received a reminder (70%). A Pearson’s chi-squared test shows that the distribution of responses across the two survey modes significantly differ (*p*-value = 0.005), which reveals a significant higher proportion of Web responses after reminder letters were used.

**Table 2:** Number of responses after first contact and after receiving reminder letters, disaggregated by mode of completion (i.e. mail or Web survey)

Region	Overall responses			Responses after first contact			Responses after reminder			p-value ^c^
	Total ^a^	Mail survey ^b^	Web survey ^b^	Total ^a^	Mail survey ^b^	Web survey ^b^	Total ^a^	Mail survey ^b^	Web survey ^b^	
Anholt	22	15 (68)	7 (32)	20	14 (70)	6 (30)	2	1 (50)	1 (50)	0.005
Keldsnor	61	51 (84)	10 (16)	31	28 (90)	3 (10)	30	23 (77)	7 (23)	
Lindet	201	155 (77)	46 (23)	119	96 (81)	23 (19)	82	59 (72)	23 (28)	
Sundeved	782	570 (73)	212 (27)	479	363 (76)	116 (24)	303	207 (68)	96 (32)	
Total	1066	791 (74)	275 (26)	649	501 (77)	148 (23)	417	290 (70)	127 (30)	

^a^ Results are given in number of respondents

^b^ Results are given in number (percentage) of respondents

^c^ Pearson’s Chi-squared test of proportions was used to compare the number of total responses after the first contact and after reminder across the two survey modes

### Self-selection analysis

Since the participants of this study had the option to self-select them into the Web or mail modes, we carried out a self-selection analysis consisting in comparing the proportion of Web survey respondents and mail respondents in each of the study regions, as well as the level of air pollution that both samples were exposed to (Table 3). Results showed that the region where the individuals live and the levels of air pollution they are exposed to were not associated with the choice of one survey mode over the other one.

**Table 3:** Self-selection bias analysis comparing the number and proportion of respondents from each study region and residential air pollution exposures for web survey respondents vs. mail survey respondents

	Web survey respondents	Mail survey respondents	p-value
Region ^a^			0.185 ^c^
Anholt	7 (2)	15 (2)	
Keldsnor	10 (4)	51 (6)	
Lindet	46 (17)	155 (20)	
Sundeved	212 (77)	570 (72)	
Residential exposure ^b^			
NO_2_ (μg/m^3^)	4.41 ± 0.53	4.42 ± 0.56	0.652 ^d^
PM_2.5_ (μg/m^3^)	10.92 ± 0.31	10.94 ± 0.27	0.466 ^d^
NH_3_ (μg/m^3^)	2.34 ± 0.86	2.28 ± 1.01	0.310 ^d^

^a^ Number of individuals (percentage)

^b^ Mean ± standard deviation

^c^ Pearson’s Chi-squared test of proportions

^d^ Two sample t-test

### Socio-demographic characteristics of web and mail survey respondents

From the data obtained by the municipalities at the year the study has started (i.e. 2014), the average age of the entire adult population (> 18 years old) living in three of the study regions (Keldsnor, Lindet and Sundeved) was 53.2 ± 18.4 years, and 49.9% of them were female. Unfortunately, data for Anholt was not available. The average age for the population at each of the regions were 57.7 ± 17.2, 53.1 ± 19.2 and 52.9 ± 18.2 for Keldsnor, Lindet and Sundeved, respectively. The average age of residents living in Lindet and Sundeved was not significantly different (*p*-value = 0.522), whereas Keldsnor’s population was significantly older than both of them (*p* <  0.0001). The percentage of female residents at each of the regions was 47.2, 49.9 and 50.1% for Keldsnor, Lindet and Sundeved, respectively. We found no significant differences in sex distribution between the regions at a confidence level of 95%.

The demographic characteristics (i.e. sex and age) of the total sample of individuals living in these three study regions were compared to respondents’ data (Table 4). A Pearson’s Chi-squared test of proportions showed no significant differences in sex distribution between respondents and the general sample (*p*-value = 0.371). However, we found the general population to be significantly younger than respondents after we carried out a two sample t-test (*p* < 0.0001).

**Table 4:** Demographic characteristics (i.e. sex and age) of the total sample of individuals living in these three study regions in comparison to respondents’ characteristics

	All sample ^a^	Respondents	p-value
Sex ^b^			0.371 ^d^
Male	11,496 (50)	518 (49)	
Female	11,456 (50)	546 (50)	
Age ^c^			
(years)	53.2 ± 18.4	59.3 ± 14.5	< 0.0001 ^e^

^a^ Data obtained by the municipalities at the year the study has started (i.e. 2014) for the entire adult population (> 18 years old) living in three of the study regions (Keldsnor, Lindet and Sundeved)

^b^ Number of individuals (percentage)

^c^ Mean ± standard deviation

^d^ Pearson’s Chi-squared test of proportions

^e^ Two sample t-test

When comparing socio-demographic characteristics of mail and Web participants, we found significant differences for most of the questionnaire items, except for smoking status and childhood living environment (Table 5). The percentage of female participants was higher for mail survey responses (*p* < 0.0001) while the average age of those who opted for Web surveys was statistically lower (*p* < 0.0001). Web participants stayed longer periods outside home (*p* = 0.010) and had higher education (*p* = 0.002). Regarding their job situation, a higher percentage of Web participants were employed at the moment (*p* < 0.0001). Besides, given the high proportion of missing data, we also analyzed whether respondents have provided information when asked about their job position, and observed that Web respondents were more likely to answer this question (*p* = 0.019). On the other hand, smoking status and childhood living environment were not significantly different between Web and mail survey respondents.

**Table 5:** Comparison between socio-demographic characteristics of mail and Web survey respondents

Socio-demographic characteristics ^a^		All respondents	Mail	Web	p-value ^b^
Sex	Male	518	352 (45)	166 (61)	< 0.0001
	Female	546	438 (55)	108 (39)	
	Missing	2	1	1	
Age (years)		59.3 ± 14.5	60.8 ± 14.9	55.1 ± 12.7	< 0.0001
	Missing	6	3	3	
Smoking status	No smoker	511	370 (47)	141 (52)	0.403
	Current smoker	158	118 (15)	40 (14)	
	Previous smoker	351	263 (34)	88 (32)	
	Passive smoker	38	32 (4)	6 (2)	
	Missing	8	8	0	
Childhood living environment	Town	223	168 (21)	55 (20)	0.192
	Village	428	305 (39)	123 (46)	
	Countryside	402	308 (40)	94 (34)	
	Missing	13	10	3	
Years living in the region		35.3 ± 20.7	36.3 ± 21.0	32.4 ± 19.6	0.006
	Missing	19	15	4	
Period outside of home	Short period (less than 14 days)	405	317 (41)	88 (32)	0.010
	Medium period (14–55 days)	553	402 (51)	151 (55)	
	Long period (56 days or more)	98	63 (8)	35 (13)	
	Missing	10	9	1	
Education	Elementary school	174	149 (19)	25 (9)	0.002
	High school	32	26 (3)	6 (2)	
	College	316	224 (29)	92 (34)	
	Short or medium higher education (1–4 years)	425	309 (39)	116 (42)	
	Long higher education (≥ 5 years)	109	74 (10)	35 (13)	
	Missing	10	9	1	
Job situation	Employed	533	350 (44)	183 (66)	< 0.0001
	Unemployed	16	11 (2)	5 (2)	
	Out of job market	513	427 (54)	86 (32)	
	Missing	4	3	1	
Job position	Informed	966	707 (89)	259 (94)	0.019
	Not informed	100	84 (11)	16 (6)	

^a^ Results are given in mean ± standard deviation for continuous variables (i.e. age and years living in the region) and number (%) for categorical variables

^b^ Determined from two sample t-test (in case of continuous variables) or Pearson’s Chi-squared test (in case of categorical variables)

### Health symptoms reported by web and mail survey respondents

The prevalence of all health symptoms (except for runny nose) was higher for participants who answered mail surveys in comparison to those who opted for the Web version (Table 6). Even after adjusting for potential confounders (i.e. age, sex, education, region where the person lives (i.e. Anholt, Keldsnor, Lindet and Sundeved) smoking status, childhood living environment, presence of children at home, period spent outside home and job situation), results obtained by logistic regression models show that the odds of reporting “increased frequency” of eyes irritation, cough and hoarseness was higher for mail respondents than Web respondents (*p*-value< 0.05). In the case of blocked nose, throat irritation and bodily pain, the differences were marginally significant (*p*-value< 0.1). For the other symptoms, the differences between the two survey modes were not statistically significant.

**Table 6:** Increased health symptom frequency for mail and Web survey respondents

Health Symptoms	% Mail ^a^	% Web ^a^	Crude OR [95%CI] ^b,c^	Adj. OR [95%CI] ^c,d^
Nose irritation	22.0	19.6	1.20 [0.85–1.69]	1.28 [0.89–1.85]
Blocked nose	25.8	24.0	1.15 [0.83–1.58]	1.39 [0.98–1.96]*
Runny nose	32.1	33.1	1.00 [0.75–1.34]	1.17 [0.85–1.60]
Chest wheezing	9.4	9.1	1.07 [0.66–1.72]	1.19 [0.71–2.01]
Breathing problems	7.5	5.1	1.55 [0.85–2.83]	1.56 [0.81–2.99]
Eyes irritation	31.2	23.6	1.53 [1.12–2.11]**	1.46 [1.04–2.05]**
Cough	27.9	20.4	1.58 [1.14–2.21]**	1.68 [1.17–2.40]**
Throat Irritation	18.8	17.5	1.14 [0.80–1.63]	1.40 [0.95–2.06]*
Hoarseness	9.2	5.8	1.70 [0.97–2.98]*	2.00 [1.09–3.69]**
Bodily pain	34.9	30.2	1.30 [0.97–1.75]*	1.36 [0.98–1.87]*

^a^ Indicates the percentage of mail and Web survey respondents that have reported “increased frequency” occurrence of each health symptom

^b^ Indicates the odds that mail survey respondents reported increased symptom frequency in comparison to Web survey respondents

^c^ *p < 0.1; **p < 0.05

^d^ Indicates the odds that mail survey respondents reported increased symptom frequency in comparison to Web survey respondents, after controlling for age, sex, education, region where the individual lives (i.e. Anholt, Lindet, Keldsnor and Sundeved), smoking status, childhood living environment, presence of children at home, period spent outside home and job situation

### Attitude towards environmental stressors reported by web and mail survey respondents

From the results shown in Table 7, it can be seen that mail survey respondents were, in general, more likely to demonstrate negative attitudes towards environmental stressors (i.e. provide positive answers for perception, annoyance, health concern and behavior interference) than Web respondents, especially after adjusting for potential confounders. Participants who chose to use mail surveys were significantly (*p*-value < 0.1) more likely: 1) to perceive noise and environmental stressors in general; 2) to be annoyed by noise and dust; 3) to be concerned with the presence of dust; 4) to be behaviorally affected by noise, odor and environmental stressors in general.

**Table 7:** Attitude towards environmental stressors for mail and Web survey respondents

Attitude towards stressors ^a^		% Mail ^b^	% Web ^b^	Crude OR [95%CI] ^c,d^	Adj. OR [95%CI] ^d,e^
Perception	Noise	22.4	20.4	1.13 [0.80–1.58]	1.51 [1.04–2.18]**
	Odor	29.5	33.1	0.84 [0.63–1.13]	1.07 [0.77–1.47]
	Dust	6.3	4.4	1.48 [0.78–2.82]	1.75 [0.87–3.54]
	Smoke	13.3	14.8	0.87 [0.59–1.29]	0.98 [0.65–1.49]
	Vibration	7.0	9.5	0.72 [0.44–1.17]	0.71 [0.42–1.21]
	Overall	20.6	18.5	1.14 [0.80–1.62]	1.38 [0.95–2.01]*
Annoyance	Noise	24.1	22.9	1.07 [0.77–1.48]	1.43 [1.00–2.05]**
	Odor	32.2	37.1	0.81 [0.61–1.07]	0.97 [0.71–1.32]
	Dust	6.6	4.4	1.54 [0.81–2.93]	1.93 [0.94–3.97]*
	Smoke	12.5	11.3	1.13 [0.73–1.73]	1.14 [0.72–1.79]
	Vibration	7.0	7.6	0.90 [0.54–1.52]	0.93 [0.53–1.64]
	Overall	16.1	14.5	1.12 [0.76–1.65]	1.32 [0.87–2.00]
Health concern	Noise	3.5	4.0	0.88 [0.43–1.79]	1.02 [0.47–2.24]
	Odor	2.8	4.7	0.58 [0.29–1.16]	0.67 [0.31–1.46]
	Dust	4.7	2.5	1.88 [0.83–4.26]	2.03 [0.87–4.76]*
	Smoke	5.9	5.5	1.09 [0.60–1.99]	1.24 [0.66–2.33]
	Vibration	1.8	2.5	0.69 [0.28–1.73]	0.86 [0.30–2.46]
	Overall	11.9	10.2	1.19 [0.76–1.86]	1.39 [0.86–2.25]
Behavioral interference	Noise	2.5	0.4	7.11 [0.95–53.21]*	9.51 [1.23–73.47]**
	Odor	10.7	6.9	1.62 [0.97–2.72]*	1.82 [1.06–3.13]**
	Dust	1.1	0.7	1.57 [0.34–7.32]	2.00 [0.40–10.14]
	Smoke	3.2	3.3	0.96 [0.44–209]	1.07 [0.47–2.40]
	Vibration	0.1	0.4	0.35 [0.02–5.56]	0.00 [0.00 - Inf]
	Overall	14.8	9.5	1.66 [1.06–2.60]**	1.87 [1.16–3.00]**

^a^ The term “environmental stressors” are defined as global conditions of the environment (in this case, noise, odor, dust, smoke and vibration) that may adversely stimulate the central nervous system and, as stressors, require adaptation or coping measurements (Campbell, 1983)

^b^ Indicates the percentage of mail and Web survey respondents that provided positive answers (scores ≥1) for each of the outcomes (i.e. variables that indicate their attitude towards stressors)

^c^ Indicates the odds that mail survey respondents reported a negative attitude towards environmental stressors in comparison to Web survey respondents

^d^ *p < 0.1; **p < 0.05

^e^ Indicates the odds that mail survey respondents reported a negative attitude towards environmental stressors in comparison to Web survey respondents, after controlling for age, sex, education, region where the individual lives (i.e. Anholt, Lindet, Keldsnor and Sundeved), smoking status, childhood living environment, presence of children at home, period spent outside home and job situation

## Discussion

In this study we provided a statistical comparison between mail and Web survey responses obtained in a cross-sectional study conducted in four non-urban areas of Denmark. Our results revealed significant differences for the majority of socio-demographic characteristics between those two groups of respondents and showed that the use of mailed surveys increased the likelihood of reporting health symptoms and negative attitudes towards environmental stressors. We found that the majority of participants preferred to answer the mail version of the questionnaire, but the proportion of Web respondents has significantly increased after the use of reminder letters.

The first objective of the present work was to analyze the study’s response rates before and after reminder letters, and how the number of responses were distributed across survey modes (i.e. print and Web surveys) at each contact point. A greater proportion of respondents opted to use mail surveys instead of Web ones. Statistics for European countries revealed that 5% of the Danish adult population does not use internet on a daily basis [31]. The updated report from the same data source [32] showed that, in 2018, 6% of the Danish households did not have access to internet. However, the share of households without internet access is considerably larger in rural areas (9%).

Besides, responding to the printed survey was in a way more convenient to participants, since the survey was already printed and included in the initial letter. Convenience is shown to be a critical factor to engage the public to participate in decisions and contribute to future implementation of policies [33]. In fact, the use of mail surveys generally yields higher response rates than Web surveys, being still considered the preferred response method [24, 34, 35]. Within this context, Smyth et al. (2010) [8] pointed out the importance of offering mail survey alternative along with the Web mode, especially when the study is conducted in rural communities.

Our results showed a substantial increase in response rates after reminder letters were used. The use of reminder letters has been emphasized in several studies as a way to increase response rates [24, 26], and a special attention is given to Web and e-mail surveys [21, 25]. When looking at the distribution of Web and mail responses at the two contact points of this study, results revealed a significant higher proportion of Web responses after reminder letters were sent. This fact may suggest a higher impact of reminders in increasing Web survey response rates in comparison to mail response rates. This finding is in agreement with the study performed by Shih and Fan (2009) [35], who showed that the use of two reminder letters (which was the same number used in our study) was acting towards a greater increase of e-mail survey response rate in comparison to mail survey responses. However, this result may be strongly explained by the way this study was designed. When residents were first contacted, the initial letter included a printed version of the survey, together with information for the Web survey completion (i.e. URL and access code). At the second (and third) contact point, the reminders contained the Web survey information, but, due to environmental reasons, did not contain the printed version of the questionnaire. Even though we offered residents the possibility of requiring a new printed questionnaire in case they did not have it any longer, this fact has likely influenced their mode selection and therefore produced a higher proportion of Web responses.

Our second purpose was to compare the demographic characteristics of mail and survey respondents. In our case, we observed a higher and statistically significant proportion of male respondents of Web surveys in comparison to mail surveys. In agreement with our findings, several studies have demonstrated that male respondents are more inclined to choose online surveys when both online and paper-based methods are available, whereas females tend to provide their answers using a paper-based method, which is more traditional [9, 36]. Since participants were free to select their preferred response method, those who opted to use the Web survey were more likely to be familiar with technology and to use internet on a daily basis. Computer use was shown to be correlated with age, education level and employment status [37, 38], explaining why Web respondents were found to be younger and higher educated and to present a higher likelihood to be employed. The demographic differences between the two survey groups are also in agreement with previous studies [39, 40], which showed that the variation in respondents’ mode preference is significantly explained by their education level, income and age. Besides, as providing responses via mail requires time for the postage, we also speculate that this is likely more accepted by the retired population, which is generally older.

Regarding the third objective, we observed that mail respondents were more prone to report the occurrence of health symptoms and negative attitudes towards environmental stressors (i.e. measured by the frequency of perception, degree of annoyance, health concerns and behavioral interference due to noise, odor, dust, smoke and vibration) even after adjusting for socio-demographic characteristics. The reason why we found statistical differences in terms of responses provided by each survey mode is still unclear. Significant measurement changes are commonly found when interview approaches (e.g. telephone and personal interview) are compared to visual approaches (e.g. mail and Web questionnaires), but previous studies have not found significant differences in responses when mail and Web responses are compared [1, 18].

We are aware of the limitations in our study. In general, our survey had a low response rate (35%). Different factors could have resulted in a low overall number of responses [21, 41]. First, the content and the topic of the survey, since in our initial contact with selected residents, we have introduced the survey as a mean to assess health and quality of life and to also investigate environment conditions in different regions of Denmark. This topic was possibly not attractive to many residents, due to an idealization of rural environments (i.e. commonly referred as the “rural idyll”) as a happier, healthier and less problematic place to live in comparison to urban areas [42]. We have also not offered any incentive for participation, which likely has impacted on the response rate obtained.

Second, the length of the survey that, although it was demonstrated in the literature to have more influence on paper-based surveys, it is typically inversely proportional to response rates regardless of the survey mode [26, 43, 44]. Third, the generic mode in which we have contacted the residents (i.e. letters were addressed “to the householder”), instead of making use of personalized letters, might have also decreased the rate of responses [11]. Accordingly, the use of personalized mail surveys was found to have even greater effects in increasing response rates of studies conducted in rural areas than urban areas [45]. On the other hand, some factors regarding the administration of our survey may have also contributed to increase the response rate of this study. Some examples of positive influencing factors can be the survey sponsorship, since surveys sponsored by research and government agencies generally yield higher response rates in comparison to commercial organization, as well as the use of mixed mode surveys and reminder letters [36].

Our study is still subject to self-selection bias, as the participants have self-selected themselves into the Web or mail survey mode. This issue may have potentially affected our results, as our samples can no longer be described as random. However, an analysis on the distribution of the responses for each survey mode in terms of the region where individuals lived and their exposure to environmental stressors showed no statistically significant differences between them. Besides, the mode selection was likely influenced by the study design, since the printed version of the questionnaire was only attached to the first letter received by the residents.

In our study, we could not fully assess whether the respondents were representative of the full population, since it is not possible to obtain detailed socio-demographic and self-reported data for all residents. However, when basic demographics (i.e. age and sex) of respondents were compared to the full sample from which they were drawn, we found respondents to be significantly older than the overall residents. There was no previous hypothesis in relation to this fact, as there is a general lack of consistency in the literature regarding the relationship between age and response rate [46].

Our study was restricted to non-urban areas, which may also influence the demographics as some studies have shown that rural residents may be significantly older and have lower educational levels [47–50]. Therefore, the conclusions should not be generalized to urban areas. In our study we did not evaluate the advantages of using mixed-mode surveys compared to using a standard single-mode approach (i.e. either mail or Web response options). A similar study has been previously conducted by Blanes-Vidal et al. (2012a, b, 2014) [51–53], in which only mail survey mode was offered to residents of six Danish non-urban areas (including the ones that were analyzed in our study). However, since the survey used in our study differs considerably from the former one, we believe the responses obtained from the two studies should not be compared.

## Conclusions

In this work, we have provided a statistical comparison between simultaneous mixed-mode survey responses collected via mailed (i.e. paper) and Web methods obtained from a cross-sectional study in non-urban areas of Denmark. One of our research objectives was to compare the distribution of mail and Web responses before and after residents received a reminder letter. We have found that the distribution of responses across the two survey modes significantly differ, showing a significant higher proportion of Web responses after reminder letters were used. Our findings suggest that the use of mail and Web surveys may produce different responses in terms of self-reported health symptoms and negative attitudes towards environmental stressors, which should be carefully considered when designing a survey study. On the other hand, given that the overall characteristics for mail and Web survey respondents differ, the use of mixed-mode approaches may provide important advantages by reaching different groups of respondents and consequently increasing response rates. Therefore, despite the challenges arisen from the mode effect, the use of mixed-mode surveys seems to be an advantageous option for studies conducted in rural communities.

## Supplementary information

**Additional file 1.** Translated version of the questionnaire used in this study.

## Data Availability

The datasets used and/or analyzed during the current study are available from the corresponding author on reasonable request.

## Abbreviations

CI: Confidence interval
NH_3_: ammonia
NO_2_: nitrogen dioxide
OR: Odds ratio
PM_2.5_: fine particulate matter
RR: Response Rate
