# Supplementary material for: Self-reported data in environmental health studies: mail vs. web-based surveys
Source: BMC Med Res Methodol. 2019 Dec 12;19:238. doi: 10.1186/s12874-019-0882-x (PMC6909640; doi:10.1186/s12874-019-0882-x)
Supplement: Supplementary file 1 — Additional file 1. Translated version of the questionnaire used in this study. [file 12874_2019_882_MOESM1_ESM.docx]

**Questionnaire**

Participants must be at least 18 years old.

Add a cross to the small circle О , or fill in the blank spaces

All information is processed confidentially and anonymously. It is important for the quality and usefulness of the study that all questions are answered.

О **Jeg er indforstået med de betingelser, som er beskrevet under "informeret samtykke".**

| Section 1: Background information  This section includes questions about your personal characteristics and general habits |
| --- |

| 1. How old are you? | | | | | | | | |  ____________ years old | | | | | | | | | |
| --- | --- | --- | --- | --- | --- | --- | --- | --- | --- | --- | --- | --- | --- | --- | --- | --- | --- | --- |
| 2. What is your sex? | | | | | | | | | О Female | | | | О Male | | | | | |
| 3. Do you smoke cigarettes? | | | О Yes, every day О Yes, but not everyday О No, but I have smoked before | | | | | | | | | | | | | | О No, I have never smoked | |
| 4. Do you live with any person that smokes regularly in the house? | | | | | | | | | О No | | | | О Yes | | | | | |
| 5. Where did you grow up? | | | | | | | | | О Large city | | | О Village | | | | О Countryside | | |
| 6. For how long have you lived in this region?  *(Not necessarily in the same residence)* | | | | | | | | | | | | | | | | __________ years | | |
| 7. How many people usually live in your house, besides you? How old are they? | | | | | | | | | | | | | | | | | | |
| ________________________________________________________________________________________ | | | | | | | | | | | | | | | | | | |
| 8. For the last 12 months, how many days have you stayed away from your house (have not slept at your house)? | | | | | | | | | | | | | | | | | | |
| О Less than 14 days | | | О 14-27 days | | | О 28-55 days | | | | О 56-111 days | | | | О 112 days or more | | | | |
| 9. What is your education level? | | | | | | | | |  | | | | | | | | | |
| О Elementary school | | О High school | | | О Professional education | | | О Short or medium higher education (1-4 years) | | | | | | | О Long higher education (5 years or more) | | | |
| 10. What is your work situation? | | | | | | | | |  | | | | | | | | | |
| О Self-employed | О Employee | | | О Unemployed | | | О Under education | | | | О Outside the job market | | | | | | | О Others (e.g. housewives) |
| 11. What is your job title? | | | | | | | | | __________________________________ | | | | | | | | | |

| Section 2: Environment  This section are about your perception of the environment in which your house is located |
| --- |

| 1. Within the past two years, have you perceived in your house (inside or near): | | | | | No/Very rarely | | | | | | Yes, several times in a year | | | | | | Yes, several times in a month | | | | Yes, several times in a week | | | | | | | | Yes, every day | | | | 2. In which period of the day do you perceive it the most? | | | | | | | | | | | | |
| --- | --- | --- | --- | --- | --- | --- | --- | --- | --- | --- | --- | --- | --- | --- | --- | --- | --- | --- | --- | --- | --- | --- | --- | --- | --- | --- | --- | --- | --- | --- | --- | --- | --- | --- | --- | --- | --- | --- | --- | --- | --- | --- | --- | --- | --- |
|  |  |  |  |  |  |  |  |  |  |  |  |  |  |  |  |  |  |  |  |  |  |  |  |  |  |  |  |  |  |  |  |  | Morning | | | | Afternoon | | | | | | | | Evening |
| Noise  Unpleasant odor  Dust  Smoke  Vibration | | | | | | О  О  О  О  O | | | | | О  О  О  О  O | | | | | | О  О  О  О  O | | | | О  О  О  О  O | | | | О  О  О  О  O | | | | | | | | О  О  О  О  O | | | | | О  О  О  О  O | | | | | О  О  О  О  O | | |
| 3. Within the past two years, have you felt annoyed by (inside or near your house): | | | | | | | | Not annoyed | | | | A little bit annoyed | | | | | | | Annoyed | | | Very annoyed | | | | | Extremely annoyed | | | | | | | **4. When is it more pronounced?** | | | | | | | | | | | |
|  |  |  |  |  |  |  |  |  |  |  |  |  |  |  |  |  |  |  |  |  |  |  |  |  |  |  |  |  |  |  |  |  |  | Spring Summer Autumn Winter | | | | | | | | | | | |
| Noise  Unpleasant odor  Dust  Smoke  Vibration | | | | | | | | О О  О О  О О  О О  О О | | | | | | | | | О  О  О  О  O | | | | О  О  О  О  O | | | | | О  О  О  О  O | | | | | О О  О О  О О  О О  О О | | | | | | | | | | О О  О О  О О  О О  О О | | | | |
| 5. What are their sources? | | | Traffic | | | | Industry | | | | | | Livestock activities | | | | | | | Slurry spreading | | | | | | | | Wind turbines | | | | | | | Unknown | | | | | Others  | | | | | |
| Noise  Unpleasant odor  Dust  Smoke  Vibration | | | О  О  О  О | | | | О  О  О  О | | | | | | О  О  О  О | | | | | | | О  О  О  О  О | | | | | | | | О  О  О  О  О | | | | | | | О  О  О  О | | | | _____________  _____________  _____________  _____________ | | | | | | |
|  |  |  | О | | | | О | | | | | | О | | | | | | |  |  |  |  |  |  |  |  |  |  |  |  |  |  |  | О | | | | _____________ | | | | | | |
| 6. Are you worried some of the following conditions in your home can harm your health? (Mark all that apply) | | | | | | | | | | | | | | | | | | | | | | | | | | | | | | | | | | | | | | | | | | | | | |
| О Yes, noise | О Yes, odor | | | | | | | | О Yes, dust | | | | | | | | | О Yes, smoke | | | | | О Yes, vibration | | | | | | | О Yes, other condition:  _________________ | | | | | | | | | | | | | | О No | |
| 7. Are there conditions that prevent you from opening your windows or doing outdoor activities (for example barbecue) as you would like? (Mark all that apply) | | | | | | | | | | | | | | | | | | | | | | | | | | | | | | | | | | | | | | | | | | | | | |
| О Yes, outside noise | | О Yes, outside odor | | | | | | | | О Yes, outside dust | | | | | | О Yes, outside smoke | | | | | | | | О Yes, outside vibration | | | | | | О Yes, other condition:  _________________ | | | | | | | | | | | | | | О No | |
| 8. If you compare the odors near your house that you used to experience 5 years ago, are they smaller or larger now? | | | | | | | | | | | | | | | | | | | | | | | | | | | | | | | | | | | | | | | | | | | | | |
| О Much larger (+50% or more)  О Larger (between +10% and +50%) | | | | | | | | | | | | | | О Same (between -10% and +10%)  О Smaller (between -10% and -50%) | | | | | | | | | | | | | | | | | | О Much smaller (-50% or more) | | | | | | | | | | | | | |
| 9. Are pesticides being applied around your home (in an area up to 100 meters from your home)? | | | | | | | | | | | | | | | | | | | | | | | | | | | | | | | | | | | | | | | | | | | | | |
| О Yes, in about 100% of the area around my house | | | | О Yes, in about 75% of the area around my house | | | | | | | | | | | О Yes, in about 50% of the area around my house | | | | | | | | | | О Yes, in about 25% of the area around my house | | | | | | | | | | | О No, no pesticide is applied | | | | | | О I don’t know | | | |

| Section 3: Health and well-being  This section asks you to provide information on your current health and daily activities, diagnosed and chronic diseases and health of any children who lives in your home on a regular basis |
| --- |

| Section 3.1: Current health and daily activities |
| --- |

| 1. Do you agree with the following statements? | Yes | | No | |
| --- | --- | --- | --- | --- |
| a. My health is good |  | О | | О |
| b. I believe my health is becoming worse |  | О | | О |
| c. I suffer from breathing problems, sleep disturbance or bad appetite |  | О | | О |
| d. I have an stressful every-day life |  | О | | О |
| e. My physical health or emotional problems disturb my social activities |  | О | | О |
| f. I go for a walk or practice other exercises in the local area |  | О | | О |
| g. I can observe from my windows: |  |  | |  |
| …any type of livestock activity |  | О | | О |
| …traffic |  | О | | О |
| …industry |  | О | | О |
| …slurry application |  | О | | О |
| …wind turbines |  | О | | О |
| …crops and vegetation |  | О | | О |
| h. During the last month |  |  | |  |
| …I felt myself full of energy |  | О | | О |
| …I have been very nervous |  | О | | О |
| …I have been very calm and peaceful |  | О | | О |
| …I have been tired |  | О | | О |
| …I have been anxious |  | О | | О |

| 2. Within the last month: | | | | |
| --- | --- | --- | --- | --- |
|  | Better than usual | Same as usual | Worse than usual | Much worse than usual |
| a. Could you concentrate on what you were doing? | О | О | О | О |
|  | Not at all | Not more than usual | A bit more than usual | Much more than usual |
| b. Did you lose sleep because of concern? | О | О | О | О |
| c. Did you feel yourself constantly under pressure? | О | О | О | О |
| d. Did you feel it was difficult to cope with problems? | О | О | О | О |
| e. Did you feel you were worthless? | О | О | О | О |
| f. Did you feel unhappy and depressed? | О | О | О | О |
| g. Did you feel you self-esteem was reduced? | О | О | О | О |
|  | More than usual | Same as usual | Less than usual | Much less than usual |
| h. Did you feel you play a useful role every day? | О | О | О | О |
| i. Did you feel you have been able to make decisions? | О | О | О | О |
| j. Were you able to enjoy your daily activities? | О | О | О | О |
| k. Were you able to identify your problems? | О | О | О | О |
| l. Did you feel happy considering everything? | О | О | О | О |

| 3. Within the past 2 years, have you experienced? | Yes, daily | Yes, several times in a week | Yes, several times in a month | Yes, several times in a year | No/very rarely |
| --- | --- | --- | --- | --- | --- |
|  |  |  |  |  |  |
| ... itching, dryness or irritation in the eyes? | О | О | О | О | О |
| ... itching, dryness or irritation in the nose? | О | О | О | О | О |
| … blocked nose? | О | О | О | О | О |
| … runny nose? | О | О | О | О | О |
| ... nausea? | О | О | О | О | О |
| ... cough? | О | О | О | О | О |
| ... chest wheezing? | О | О | О | О | О |
| ... other type of breathing difficulties? | О | О | О | О | О |
| ... unnatural fatigue? | О | О | О | О | О |
| ... headache? | О | О | О | О | О |
| ... difficulties concentrating? | О | О | О | О | О |
| … depression? | О | О | О | О | О |
| ... dizziness? | О | О | О | О | О |
| … tinnitus? | О | О | О | О | О |
| ... sleeping problems? | О | О | О | О | О |
| … diarrhea? | О | О | О | О | О |
| … hoarseness? | О | О | О | О | О |
| … pain or irritation in the throat? | О | О | О | О | О |
| … bodily pain? | О | О | О | О | О |

| Section 3.2: Diagnosed symptoms and chronic diseases |
| --- |

| 1. Within the last 2 years, have you had any acute (not chronic) respiratory problem (such as bronchitis, influenza, pneumonia or other infection)? | If answered ”yes”: Which problem? |
| --- | --- |
| О Yes О No О I don’t know __­­­­__________________________________ | |

| 2. Do you have any of the following diseases? (Mark all that apply)? | | | |  | |
| --- | --- | --- | --- | --- | --- |
| О Asthma | О Chronic bronchitis | О Allergic rhinitis | О Pulmonary disease | | |
| О Cardiovascular diseases | О High blood pressure | О Any other disease in the respiratory tract | | |  |

| 3. Are you allergic to anything? | If answered ”yes”: To what? How old were you when you were diagnosed? |
| --- | --- |
| О Yes О No О I don’t know ___­­­­­­­­­___________________________________________________­­­­___­­___ | |

| 4. Do you have any other chronic disease? | If ”yes”: Which one? How old were you when you were diagnosed? |
| --- | --- |
| О Yes О No О I don’t know ___­­­­­­­­­_________________________________________________­­­­__ | |

| Section 3.3: Health of any children who lives in your home on a regular basis  (If there not any children (under 18 years old) in the house, you can move to questions 8 and 9). |
| --- |

|  | Child 1 | Child 2 | Child 3 | Child 4 |
| --- | --- | --- | --- | --- |
| 1. What if the sex of each child? | О Female  О Male | О Female  О Male | О Female  О Male | О Female  О Male |
| 2. What is the age of each child | ______ years | ______ years | ______ years | ______ years |
| 3. What is the general health of each child? | О Very good  О Good  О Poor | О Very good  О Good  О Poor | О Very good  О Good  О Poor | О Very good  О Good  О Poor |
| 4. Have the child had problems with (in the last year) | **Yes No** | **Yes No** | **Yes No** | **Yes No** |
| … whistling or wheezing in the chest? | О О | О О | О О | О О |
| … runny or blocked nose when he/she had not a cold or flu at the moment? | О О | О О | О О | О О |
| … itching, dryness or irritation in the eyes when he/ she had not a cold or flu at the moment? | О О | О О | О О | О О |
| … cough when he/she had not a cold or flu at the moment? | О О | О О | О О | О О |
| 5. Has this child ever had asthma? | О О | О О | О О | О О |
| 6. Does he/she still have asthma? | О О | О О | О О | О О |
| 7. Has a doctor ever said that this child had | **Yes No** | **Yes No** | **Yes No** | **Yes No** |
| …Hay fever? | О О | О О | О О | О О |
| …Allergy? If so, which type of allergy? | О О  _________ | О О  _________ | О О  _________ | О О  _________ |

| 8. Feel free to comment or write comments on this questionnaire! | | |
| --- | --- | --- |
|  | | |
| 9. | О | **VERY IMPORTANT!**  **I agree with the terms described under "Informed consent for participation".** |

**Thank you very much for answering the questionnaire!**

Informed consent for participation

I have read and I understood the information about the study.

I have been informed that **my participation in the study is voluntary** and that **it will not have any consequences or disadvantages if I decide to not participate in it**.

I hereby agree to participate in the investigation and that my data will be collected and analyzed in an **anonymously and confidentially manner**. Data will only considered by the research team carrying out the study and **will only be used for research purposes.**

This consent can be terminated at any time by sending an email to vbv@mmmi.sdu.dk or mlca@mmmi.sdu.dk

The survey was approved by the Danish Data Protection Agency.
